# Conventional Versus Osseodensification Drilling in the Narrow Alveolar Ridge: A Prospective Randomized Controlled Trial

**DOI:** 10.7759/cureus.56963

**Published:** 2024-03-26

**Authors:** Mohanasatheesh Shanmugam, Mohan Valiathan, Anitha Balaji, Angelin Fiona Jeyaraj Samuel, Rudra Kannan, Vishnu Varthan

**Affiliations:** 1 Periodontics, Sree Balaji Dental College and Hospital, Chennai, IND

**Keywords:** resonance frequency analysis (rfa), bone density, narrow ridge, osseodensification, implant drills

## Abstract

Background

Conventionally, undersized osteotomies were used to increase initial bone-to-implant contact to achieve primary stability in implantology. This is particularly evident in regions with low bone density. The potential for severe bone compression and ischemia poses a challenge to secondary stability. Instead, lateral bone compaction is caused by the idea of osseodensification. Research on the potential benefits of this method for narrow ridges is lacking. This study aimed to determine if the osseodensification drilling technique affects primary stability and how much the alveolar ridge expands following implant site preparation.

Methodology

A total of 30 participants aged 20 to 80 years were included in this randomized controlled clinical investigation. Each participant was randomly assigned to one of the following two groups: one that received standard drill preparation, and another that received osseodensification drill preparation. Implant stability using implant stability quotient values, crest width, apical width (5 mm from crest), and bone density were assessed both before and after six months using cone-beam computed tomography.

Results

Osseodensification impacted the width at the apex (5 mm from the crest) and radiographic bone density, adding to the quality, but did not affect implant stability and crestal width after osseointegration. The mean difference in conventional and osseodensification groups was 0.46 and 0.68 mm, respectively, concerning the crestal width. Moreover, the mean difference was 0.74 and 0.58 mm for conventional and osseodensification groups, respectively, concerning the width at the apex (5 mm from the crest).

Conclusions

This study demonstrates that the osseodensification process increased both the radiographic bone density and the width at the apex, demonstrating that osseodensification drilling techniques allow for the placement of implants with larger diameters in narrow alveolar ridges.

## Introduction

Despite available treatments, tooth loss remains common. Factors such as trauma, periodontal disease, and failed endodontic treatment contribute to tooth loss in 69% of 35-44-year-olds. By age 74, one in four individuals globally will be fully edentulous [1]. Dental implants are now the preferred method for replacing missing teeth [2].

Dental implants are essentially artificial roots that replace natural ones. The pros of the procedure include a low sensitivity of neighboring teeth, maximum prevention of bone resorption at the edentulous site, and a high success rate (above 97% for 10 years). Globally, between 100,000 and 300,000 dental implants are implanted annually, and this figure is rising [3,4].

Factors such as bone density, surgical technique, and implant configuration play a crucial role in determining the primary stability of dental implants [5-8]. During the healing phase, mechanical stabilization of the implant screw is necessary to prevent dislodgement [9]. Friction between the implant and surrounding bone is key and can be evaluated by comparing insertion torque and bone density [10]. Higher insertion torque results in greater initial bone-to-implant contact, leading to improved primary stability [11]. Ottoni et al. (2005) demonstrated an inverse correlation between failure rate and insertion torque [12].

To make room for the implant fixture, the standard drilling method often involves excavating bone through the subtracting drilling technique. Current evidence mostly disregards osteotomy for the aforementioned objective [13-15].

To achieve primary stability, the initial bone-to-implant contact is maximized using normally undersized osteotomies. This is particularly the case in places where bone density is low [16,17]. However, this could lead to severe compression of the bones and ischemia, which would be a concern with secondary stability. To overcome this problem, osteotomies utilizing piezosurgery were developed and implemented. By compressing the bone on both sides, bone compactors improved primary stability. There was a need for a more recent method that was both more effective and less harmful to bone because all the previous methods had drawbacks [18].

The term osseodensification was first used by Huwais and Meyer (2017) [14]. The goal was to create an implant osteotomy with increased bone density using autologous lateral bone compaction to preserve bone density and prepare the implant location with minimal stress. In the long run, this would boost primary stability, insertion torque, and bone-implant contact [18], leading to improved success rate and patient satisfaction.

Because of viscoelastic and plastic deformation, the cancellous bone is compressed during osseodensification, and, at the same time, the autogenous bone fragments are compacted acting as a seeding agent for osteogenesis. Additionally, it has the potential to completely alter the way preparation is done for situations involving low-density bone. Reducing the size of the osteotomy upon removal is an advantage of the osseodensification burs. The viscoelastic properties of bone contribute to this phenomenon, which is known as the springback effect [19].

Osseodensification is a technique that uses a special bur called Densah™ bur to densify bone and improve implant stability. These burs are designed to operate in a counterclockwise direction and create rolling and sliding contact. Unlike conventional drills, osteotomes caused by these burs do not shatter the bone trabeculae, leading to faster healing and better secondary implant stability. However, osseodensification cannot be performed on cortical bone or xenografts. The effectiveness of this technique in narrow ridges remains uncertain, prompting this study to evaluate the expansion of the alveolar ridge and the impact of osseodensification on implant stability.

## Materials and methods

Study design

A total of 30 individuals in need of dental implants participated in this randomized controlled clinical trial. The sample size was calculated using G*power 3.1.9.4 [20]. Participants were recruited from the outpatient clinic of the Department of Periodontology and Implantology at Sree Balaji Dental College and Hospital in Chennai, Tamil Nadu, India. Informed consent was duly acquired from all individuals involved in the study, who were aged between 20 and 80 years of both genders. Before conducting the study, the researchers obtained approval from the Research Ethics Committee of the Faculty of Dentistry, Sree Balaji Dental College and Hospital (approval number: SBDCH-IRB 22-06/09). The implant implantation procedure adhered to biosafety standards by requiring patients to undergo preoperative medical testing.

After thoroughly and clearly outlining the procedures, including any potential advantages and negative effects, all patients were asked to provide their informed consent. In addition, patients had the choice to quit at any point in the study.

Inclusion criteria

Participants eligible for the study included men and women aged 20 to 80 years who were willing to participate, had maintained adequate oral hygiene, had at least one missing tooth in both maxilla and mandible posteriors of D3-D4 bone type, and had an alveolar ridge width of 3-6 mm at the crest bone level buccolingually.

Exclusion criteria

Individuals with an edentulous region with high bone density D1 type based on cone-beam computed tomography (CBCT) findings, pregnant and lactating mothers, patients with parafunctional habits such as severe bruxism and clenching, heavy smokers who smoked more than 10 cigarettes per day, patients with medical conditions that could interfere with implant placement, and those receiving chemotherapy or radiotherapy were excluded. These patients were excluded due to the potential risks and complications that these conditions can pose to the success of dental implants and the overall safety of the participants.

Materials used

The study utilized a range of materials, including implant systems from Dentium Co. Ltd., Korea, and the Osstem Implant System. Additionally, Densah burs provided by Versah and the Osstell ISQ-Mentor device, along with smart pegs, were employed in the study. These materials were integral to the study’s methodology and findings.

**Figure 1:**
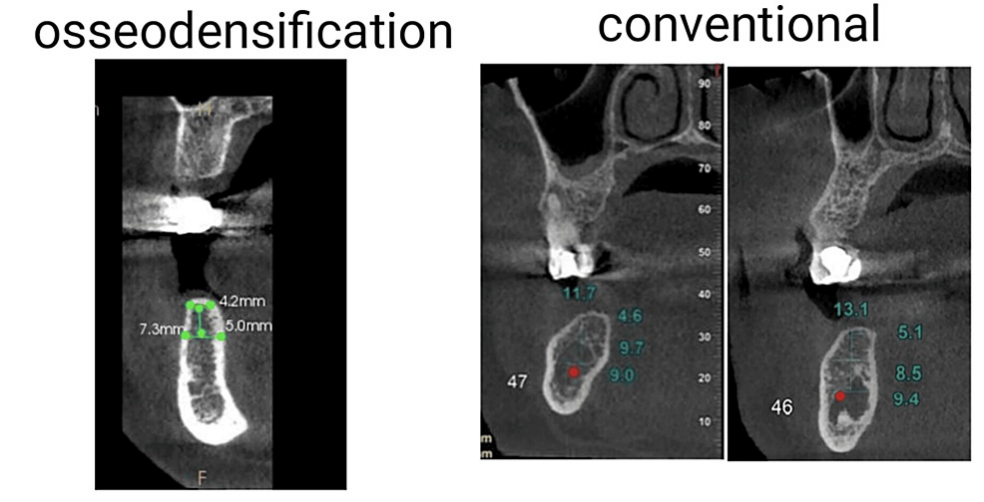
Preoperative cone-beam computed tomography showing narrow alveolar ridge.

The pre-surgical assessment was conducted following standard protocols, and preoperative 3D CBCT was performed to evaluate buccolingual width, bone quality, and height of the alveolar ridge. Buccolingual width of less than 6 mm was considered in the study (Figure 1).

Surgical phase

**Figure 2:**
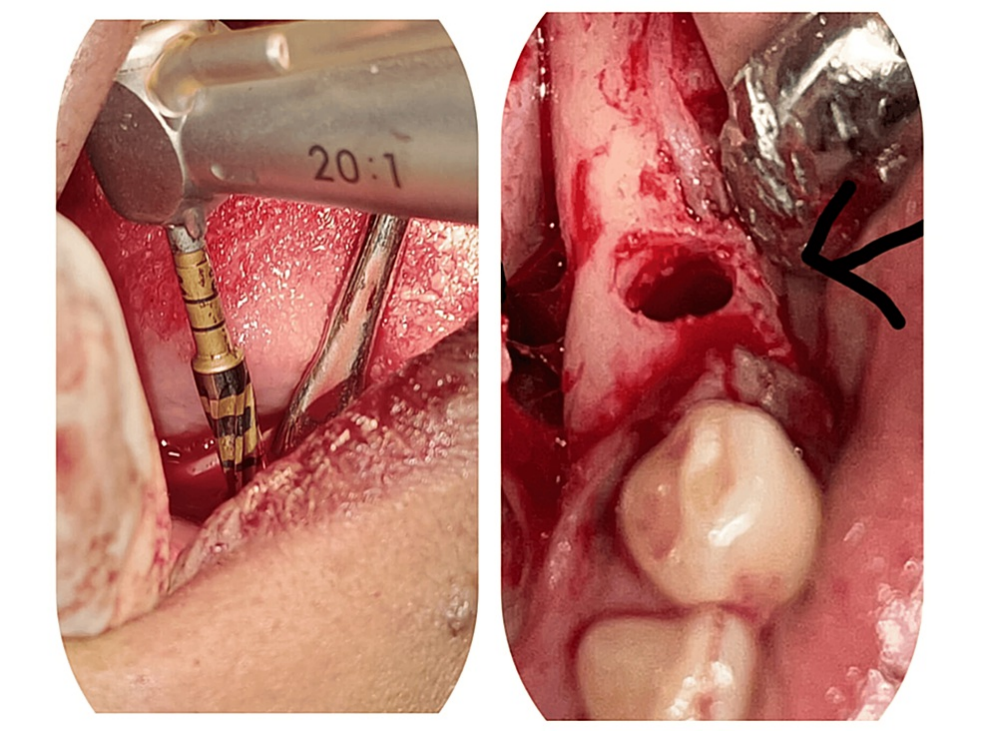
Expanded osteotomy site in narrow alveolar ridge using Densah burs.

A mouthwash containing 0.12% chlorhexidine was used to disinfect the oral cavity. At the surgical location, a local anesthetic consisting of 2% lidocaine and 1:1,000,000 epinephrine was injected. Of the 30 patients, 15 underwent traditional drilling osteotomy, and 15 underwent osseodensification (Versah bur) drilling. According to the manufacturer’s drilling protocol, sequential drilling osteotomy was performed in a clockwise direction for conventional drilling and a counterclockwise direction for osseodensification drilling (Figure 2).

**Figure 3:**
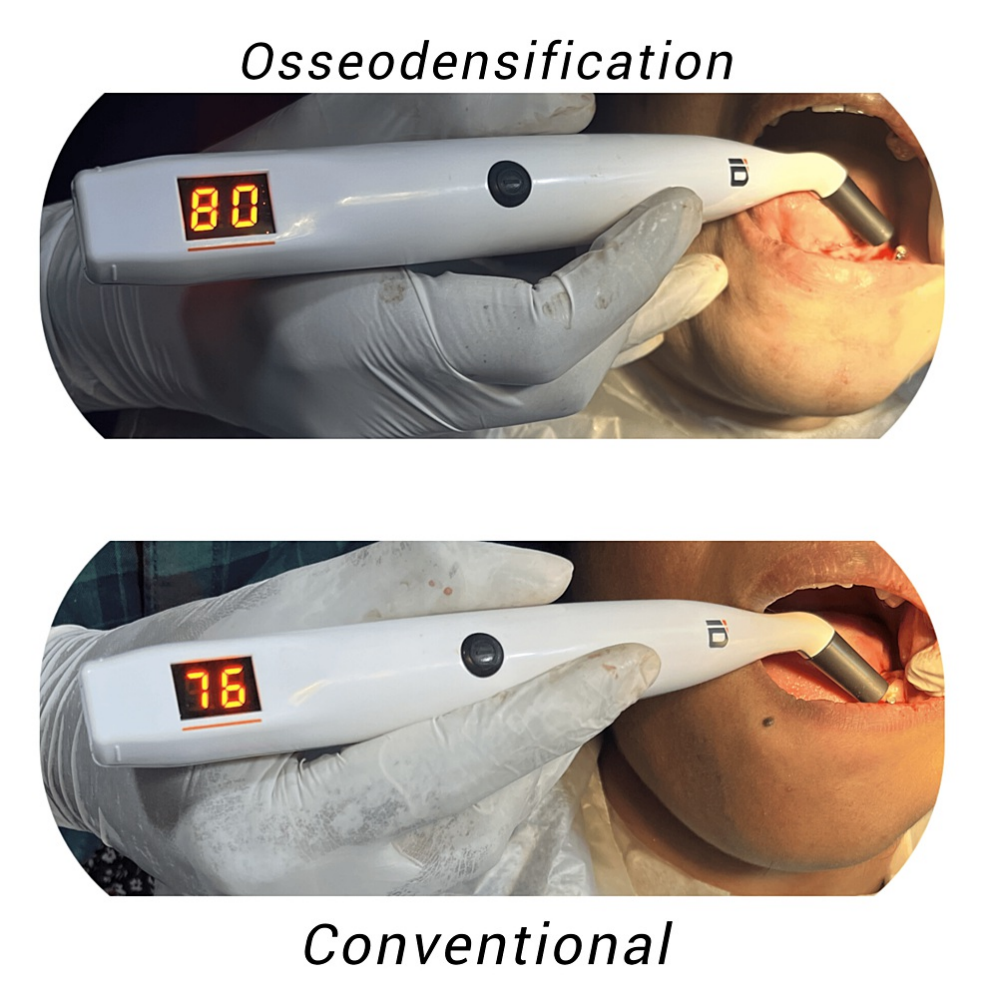
Primary implant stability quotient recorded immediately after implant placement.

Once the implants were in place, the Osstell ISQ-Mentor and a smart peg were used to record the implant stability quotient (ISQ) values (Figure 3). The flap was repositioned, and 3-0 silk sutures were used to achieve primary wound closure at the implant site.

Post-surgical phase

The patient received postoperative care following established protocols. Starting immediately and continuing throughout the next day, all patients were asked to apply cold fomentation. They were advised on the need for good dental hygiene. The doctor prescribed antibiotics and non-steroidal anti-inflammatory drugs to be taken every eight hours for five days, and for seven days, a mouth rinse containing 0.12% chlorhexidine was recommended.

The sutured wound was carefully checked for any indications of infection, such as redness, swelling, heat, pus discharge, or discomfort, and the sutures were taken out after a week. Additionally, any indicators of interruption to the wound healing process, such as dehiscence, were noted. Following surgery, the sutures were removed one week later [18].

Pre-prosthetic phase

**Figure 4:**
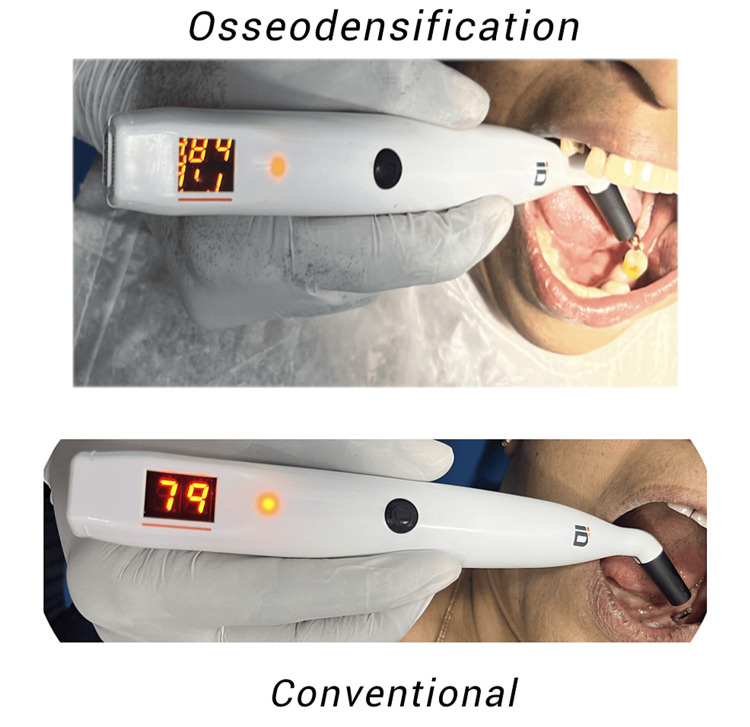
Implant stability quotient recorded after three months.

The cover screw was removed, and ISQ values were recorded to compare the biological values with primary stability values. Healing abutments were placed and the patients were asked to report after two weeks for the prosthetic phase (Figure 4). After being extracted from their respective implants, all smart pegs were chemically cleaned and placed in a sterilization pouch containing the data of all patients [19].

Final prosthesis

**Figure 5:**
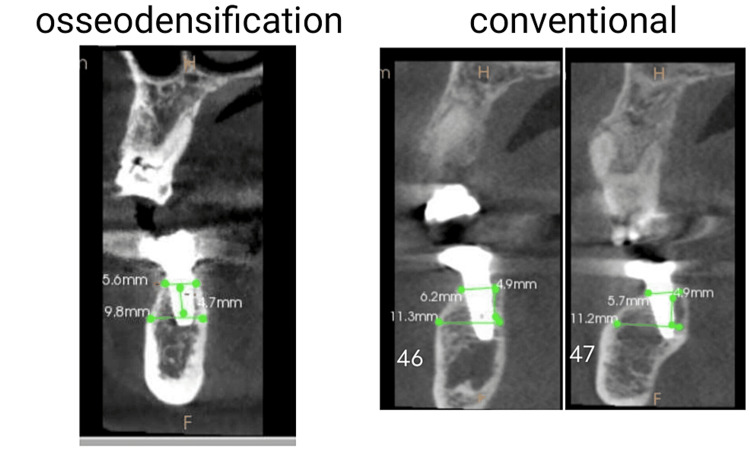
Six-month postoperative cone-beam computed tomography to evaluate alveolar ridge expansion.

An impression was taken and the prostheses were delivered to the patients between three to six months. Postoperative CBCT was performed (Figure 5).

Statistical analysis

Implant stability was assessed at baseline and after three months. Bone density, crest width, and width 5 mm at the apex from the crest were assessed at baseline and six months. R Core Team 2021 (Statistical Computing, Vienna, Austria, URL https://www.R-project.org/) was used to conduct statistical analysis, with data entered in Microsoft Excel 2010 (Microsoft Corp., Redmond, WA, USA). Mean, standard deviation, minimum, and maximum values were used to describe the data. Based on the results of the Shapiro-Wilk test for normalcy, either parametric or non-parametric tests were employed. The paired t-test was utilized for crestal width to analyze differences in parameters between two time intervals, while the Wilcoxon test was employed for all other pairings. The Mann-Whitney U test was employed to compare the groups at different time intervals.

## Results

During the six months following CBCT, there was no difference in crestal width between the conventional and osseodensification drilling groups, even though the latter approach induced increased diameter in the narrow ridge during osteotomy. However, when measured 5 mm from the apex to the crest, there was a substantial difference in the width in the osseodensification group, with the conventional group exhibiting more variability. Both sets of data demonstrated a statistically significant improvement in radiographic bone density. Both groups had a statistically significant increase in implant stability over time, as determined by ISQ values. The outcomes are presented in Table 1.

**Table 1:** The summary of values obtained in the study.

		Crest width		Apical width		Hounsfield (HU)		Implant stability quotient	
		Baseline	6 months	Baseline	6 months	Baseline	6 months	Baseline	3 months
Conventional	Mean	4.75	5.21	7.85	8.59	579.06	863.73	74.4	79.33
	SD	0.91	0.83	1.95	1.65	164.77	320.53	6.10	5.26
	Minimum	3.2	3.6	3.6	5.8	337	475	59	67
	Maximum	5.8	6.7	10.5	10.5	853	1,600	83	88
	P-value	0.09906		0.0038		0.000061		0.0010	
Osseodensification	Mean	4.24	4.92	5.61	6.20	630.21	1,186.21	70.46	78.87
	SD	1.03	1.03	1.59	1.56	254.33	282.32	8.81	5.04
	Minimum	3	3	3.6	4.2	283	833	55	69
	Maximum	5.8	6.9	10.2	10.4	1,236	1,747	84	85
	P-value	0.0006748	Paired t-test	0.002301	Wilcoxon	0.0001221	Wilcoxon	0.000714	Wilcoxon
	P-value (MWU)	0.1364	0.484	0.002986	0.002065	0.8103	0.00589	0.3281	0.8512
	Shapiro-Wilk P-value	0.544		0.005105		0.005756		0.001426	

## Discussion

This clinical trial evaluated the outcome of delayed implant placement with either osseodensification or conventional drilling techniques. The clinical and radiographical outcomes were assessed and compared at baseline and three and six months later.

Successful dental implants rely on the alveolar ridge, an important anatomical component. The extraction of a tooth from the lower or maxillary alveolar ridge often results in the loss of bone. Bone loss, or alveolar ridge resorption, can impact anywhere from 30% to 60% of the bone mass. Due to a lack of thick bone in the alveolar ridge, implant hardware placement might be challenging [21].

A minimum of 1 mm of buccal and lingual bone width relative to the implant surface is required for long-term bone coverage and implant success, as demonstrated in multiple clinical investigations. Several surgical techniques have been developed to address the issue of thin alveolar ridges. These include distraction osteogenesis, guided bone regeneration, onlay (veneer) block bone grafting with intraoral sources, and ridge splitting/expansion [22].

It has been shown in the literature that for the implant procedure to proceed smoothly, a ridge width of at least 6 mm is required, which implies at least 1-1.5 mm of bone around the implant would be needed [23].

According to Huang et al. (2020) [24], numerous biological and clinical factors impact ISQ results. Regarding radiograph bone density, implant stability, and ridge width at the crestal and apical levels, the present study compared implants placed using osseodensification to those implanted using traditional osteotomy.

Based on current evidence, keeping the crestal peri-implant bone is essential for the therapy to be successful. This is because the bone surrounding the implant provides support for the surrounding soft tissue, which is crucial for both the implant’s aesthetics and its long-term viability [25].

When comparing osseodensified and conventional osteotomy sites at three and six-month intervals, Aloorkar et al. (2022) [26] discovered no statistically significant change in crestal bone levels. Our study also concluded that there was no statistically significant difference in crestal width between the two groups after six months. Statistical analysis, however, revealed an uptick in the osseodensification group. The osseodensification group had a mean difference of 0.68 mm, while the conventional group had a difference of 0.46 mm. The finding was not statistically significant when comparing the baseline values of the two groups. Further, even after three months, there was no discernible difference between the groups. Consequently, there was no discernible contribution of osseodensification to the variance in crestal breadth, and the two groups were comparable.

The 5 mm width from the apex to the crest changed over time in both sets of data. Additionally, there was a notable difference between the groups at both the baseline and three-month marks. The conventional group had a mean difference of 0.74 mm, while the osseodensification group had a mean difference of 0.58 mm. When contrasted with the osseodensification group, the conventional group exhibited more diversity.

After reviewing 195 papers, Pai et al. (2018) [18] found that osseodensification causes an osteotomy that is smaller than what would be achieved with traditional drills. More bone density, more bone volume percentage, and more bone-to-implant contact are all outcomes of this process that enhance implant durability. According to research by Hindi and Bede 2020 [27], osseodensification causes a notable rise in peri-implant bone density, with a 60 HU increase. Osseodensification results in less bone loss compared to other surgical procedures, as demonstrated by Al-Ahmari (2022) [28] in a study of postoperative bone density.

Only a handful of studies, such as those by Barone et al. in 2003 and Hasan et al. in 2014 [29] conducted radiological assessments of bone density surrounding implants. These studies utilized the grayscale values from CBCT images taken six months after the procedure to measure bone density.

The mean differences of the two groups in radiographic bone density were 284.67 and 556 HU, respectively, indicating a statistically significant improvement. The osseodensification rise is about twice that of traditional osteotomy. There was no significant difference between conventional and osseodensification values at baseline in this parameter, but there was a significant difference between the values at three months in the two groups. Consequently, the osseodensification process played a role in the enhancement of bone density.

There is evidence that osseodensification increased primary stability [18,27]. However, there was no discernible difference between osseodensification and traditional drilling, according to Sultana et al. (2020) [30]. With a mean difference of 4.93 and 8.41 in ISQ values, respectively, there was a statistically significant improvement in stability over three months for the conventional osteotomy and osseodensification groups concerning implant stability, as determined by ISQ. The groups did not differ significantly in their three-month ISQ values, hence the change in mean ISQ was likely due to chance. Consequently, after three months, osseodensification did not affect the stability of the implants.

The osseodensification process, which aids in expanding the crest during osteotomy, showed no notable difference in the width of the crestal bone after six months when set against conventional techniques. Nevertheless, it did contribute to an increase in the bone’s width 5 mm beneath the crest, leading to improved radiographic bone density based on the Hounsfield unit measurement.

While several techniques exist to increase the width of the alveolar ridge, often necessitating over six months of waiting, the osseodensification method presents advantages. Notably, it was observed to prevent dehiscence or fenestration during this study, potentially lowering both morbidity and the cost of treatment.

Employing Densah burs, which are designed with a greater negative rake angle and operate at 800-1,200 rpm in a counterclockwise motion with adequate irrigation, creates hydrodynamic waves that compact bone particles and promote bone formation. The study noted an enhancement in primary stability above 70 Ncm for both methods, with a marginal preference for osseodensification, although this was not statistically significant.

The aims to this study were to determine whether the osseodensification bur affects primary stability at prepared sites and to evaluate the extension of the thin alveolar ridge following implant site preparation. Specifically, we wanted to determine whether Densah bur could improve primary stability in the narrow alveolar ridges. The results show that in this study performed in narrow ridges, osseodensification did not help with implant stability. The study indicated that osseodensification enhanced the bone density at the apical region and overall radiographic bone density, yet it did not significantly impact the width of the crestal bone or the stability of the implant compared to the traditional drilling approach.

The study’s limitations include a small sample size with samples from both maxillary and mandibular partially edentulous regions. A longer-term clinical trial could help address these limitations and provide more precise recommendations. Despite the current study’s constraints, no clinically relevant differences were noted between the osseodensification and conventional drilling techniques regarding ISQ values. However, the osseodensification technique did significantly enhance apical width and bone density, as measured in CBCT 5 mm apical to the crest.

## Conclusions

The osseodensification drilling method has shown a statistically significant enhancement in bone density and an increase in alveolar ridge width, particularly in the apical ridge area surrounding the implants, while maintaining primary stability with a relative increase over conventional drilling methods. The technique aids in conserving existing bone, thereby reducing the likelihood of dehiscence or fenestration. Additionally, it also eliminates the necessity for bone grafting procedures, allowing for the placement of wider implants in narrow alveolar ridges.

Hence, within the limitations of the study, the osseodensification drilling technique may offer a more efficient and less invasive option for patients requiring dental implants in narrow alveolar ridges, thus having a definite edge over the conventional drilling protocol.
